# Tumor immune microenvironment of cutaneous angiosarcoma with cancer testis antigens and the formation of tertiary lymphoid structures

**DOI:** 10.3389/fonc.2023.1106434

**Published:** 2023-04-04

**Authors:** Tetsuya Magara, Motoki Nakamura, Yuka Nojiri, Maki Yoshimitsu, Shinji Kano, Hiroshi Kato, Akimichi Morita

**Affiliations:** Department of Geriatric and Environmental Dermatology, Graduate School of Medical Sciences, Nagoya City University, Nagoya, Japan

**Keywords:** cutaneous angiosarcoma, tertiary lymphoid structures, cancer testis antigen, immune checkpoint inhibitors, next generation sequence (NGS)

## Abstract

Cutaneous angiosarcoma (CAS) is a highly malignant tumor with few effective treatments. Although the indication for immune checkpoint inhibitors such as anti-PD-1 antibodies is expected to expand, there are many unknowns regarding the tumor immune microenvironment in CAS, which is generally considered an immunologically “cold” tumor. Our previous study demonstrated that tertiary lymphoid structures (TLSs) were associated with a favorable prognosis in CAS. However, we still don’t know what the difference is between cases of TLS-rich and TLS-poor. Furthermore, the number of TLSs can vary significantly between lesions in the same case, for example, between primary and recurrence. To analyze the changes in the tumor immune microenvironment in CAS in more detail, we performed comprehensive RNA sequencing using a Next-generation sequencer (NGS). Sixty-two samples from 31 cases of CAS treated at Nagoya City University were collected. NGS and gene set enrichment analysis (GSEA) were performed on 15 samples among them. Immunohistochemistry and prognostic analysis by Kaplan-Meier method were performed on all 62 samples. NGS results showed that NY-ESO-1 (CTAG1B) was significantly upregulated in the TLS-positive cases. Immune checkpoint molecules including programmed death-1 (PD-1) and programmed death-ligand 1 (PD-L1) were upregulated in TLS-negative or TLS-low cases and seemed to associate with the suppression of TLS formation. In a comparison of primary and recurrent lesions, other cancer-testis antigens (CTAs) including XAGE-1B were significantly upregulated in recurrent lesions. The number of infiltrating CD8-positive cells and TLSs showed no significant trend between primary and recurrent lesions. However, the PD-L1 expression of tumor cells was significantly lower in recurrent than in primary lesions. Chemokines correlated with NY-ESO-1 expression were CCL21 and CXCL8, and only CCL21 correlated with the number of TLS. There was no chemokine associated with XAGE-1. NY-ESO-1 and XAGE-1 are detectable by immunohistochemistry. Although each cannot be a prognostic marker by itself, they can be a helpful marker in combination with the number of TLSs. CTAs play an essential role in forming the tumor immune microenvironment in CAS. These findings are evidence that CAS is an immunologically “hot” tumor and provides us with potential therapeutic targets and encourages the expansion of immunotherapy indications.

## Introduction

Cutaneous angiosarcoma (CAS) is a rare cutaneous malignancy with rapid proliferation and a tendency to develop lymph node and pulmonary metastases, with a very poor prognosis, and is most commonly found in the head of elderly patients. Because of the small number of cases, research has not progressed much and effective treatments are few and far between. In the last few years, there have been several reports of immune checkpoint inhibitors (ICIs) being effective in CAS cases (1–3), which is expected to be a new effective therapy. Although CAS, as well as other soft tissue sarcomas, has been considered an immunologically “cold” tumor, our previous study of 61 samples from 31 cases of CAS revealed that there are active anti-tumor immune responses with abundant immune cell infiltration, including the formation of tertiary lymphoid structures (TLSs) (4). TLSs are lymphoid follicle-like clusters of CD20-positive B cells that form near the tumor or inflammatory lesions and are typically surrounded by CD3-positive T cells. They function as a front base for antigen presentation and T-cell activation and have been reported to be useful not only as a marker of good prognosis in many cancers but also as a predictive marker of response to immunotherapy, including ICIs (5, 6). TLSs were observed in about half of the CAS cases, and those with TLS had a better prognosis than those without TLS. When ICI therapy for CAS is approved in the near future, TLSs may be useful as a predictive marker of ICI efficacy. However, we do not yet know what influences the immunological activity of CAS, including the presence or absence of TLSs, and what the tumor microenvironment is like for CAS with and without TLSs. Furthermore, the presence or absence of TLSs and their number can vary significantly between lesions in the same case, for example, between primary and recurrent lesions (7). Further investigation of anti-tumor immunity in CAS is a necessary process for the expansion of applications of ICIs, which is currently expected worldwide. Here we present the results of the analysis of 395 immune-related factors in CAS using next-generation sequencing. To our knowledge, this is the first report of comprehensive RNA sequencing in CAS.

## Materials and methods

### Patient samples

A total of 62 formalin-fixed paraffin-embedded (FFPE) samples from 31 Japanese patients histologically diagnosed with CAS based on biopsy or surgical resection samples were obtained at Nagoya City University Hospital. The cohort is summarized in **Supplementary Table 1**. This is the cohort of our previous report with one additional lymph node metastasis sample (4). Immunostaining was performed in this cohort. Comprehensive RNA sequencing using next generation sequencer (NGS) was performed on 15 samples from 7 cases selected from this cohort (**Table 1**). Patients with primary and recurrent lesions were selected, including one case with multiple recurrences. The cohort included 6 men and 1 woman with a median age of 73.29 (range 62-81) years. 6 cases (85.7%) occurred in the head and neck, and 1 case (14.3%) occurred in the trunk. According to the pathological differentiation classification, 12 samples were highly differentiated, and 3 samples were poorly differentiated. These patients were treated with one or a combination of the following: surgery, radiation therapy, taxanes, eribulin, or pazopanib.

**Table 1:** Characteristics, treatment, and immunofluorescence staining results for RNA sequenced patients.

Characteristics		Value
Cases		7
Samples		15
	Primary lesion	7 (7 cases)
	Recurrent lesion	8 (5 cases)
Age (range)		73.29 (62–81)
Sex		
	Male	6 (85.7%)
	Female	1 (14.3%)
Primary Site		cases (n=7)
	Head&Neck	6 (85.7%)
	Trunk	1 (14.3%)
	Extremity	0
Metastases (at diagnosis)		Cases (n=7)
	Lymph node metastasis	0
	Distant metastasis	0
Differentiation		Cases (n=7)
	Well-differentiated	5
	Moderately differentiated	0
	Poorly differentiated	2
Treatment		cases (n=7)
	Surgery	5 (71.4%)
	Radiation therapy	6 (85.7%)
	Interleukin-2	0
	Taxanes	6 (85.7%)
	Eribulin	2 (28.6%)
	Pazopanib	2 (28.6%)
PD-L1 expression		samples (n=15)
	Higher than average (47.4 pv)	7
	Lower than average	8
CD8 infiltration		samples (n=15)
	Positive	9
	Negative	6
The number of TLSs		samples (n=15)
	0	4
	1-4	5
	5-9	3
	10 or more	3
pv, pixel value.		

### RNA extraction and sequencing

RNA extraction and sequencing were performed as previously described (8). Tumor tissue was carefully dissected from 3 to 10 undyed FFPE tissue sections (7 µm thickness) using a scalpel blade and deparaffinized in 640 µl deparaffinization solution (Qiagen, Hilden, Germany). According to the supplier’s instructions, total RNA was refined using an AllPrep DNA/RNA FFPE Kit (Qiagen). The concentration and quality of the extracted RNA were evaluated by Qubit RNA HS Assay Kit using Qubit 3.0 Fluorometer and by a functional RNA quantitation (FRQ) assay using StepOne Plus (Thermo Fisher Scientific, Waltham, MA). RNA samples confirmed to be of sufficient quality were reverse-transcribed to cDNA samples, which were amplified using Ion AmpliSeq Library Kit 2.0 (Thermo Fisher Scientific). Ion Library TaqMan Quantitation Kit measured the concentration of library DNA samples using ABI7500FastDx (Thermo Fisher Scientific). Amplified library DNA samples were applied to the NGS using Oncomine Immune Response Research Assay (Thermo Fisher Scientific). NGS analysis was performed using the Ion S5 XL System (Thermo Fisher Scientific). Data were analyzed on the software application Transcriptome Analysis Console and Gene spring analysis (Thermo Fisher Scientific). All data were uploaded to the national center for biotechnology information (NCBI) gene expression omnibus (GEO) database (GSE203215).

### Gene set enrichment analysis

Gene set enrichment analysis (GSEA) was performed using the c5 Gene Ontology gene set collections as provided by the Molecular Signatures Database (MSigDB) (9) and GSEA software (https://www.gsea-msigdb.org/gsea/) (10).

### Immunohistochemical staining

FFPE tissue sections of 62 CAS samples were processed for indirect immunofluorescence to detect the expression of signal transduction proteins using primary antibodies: anti-NY-ESO-1 antibody (1:100, ab223498, Abcam, Cambridge, UK), anti-XAGE-1 antibody (1:130, ab134805, Abcam), anti-PD-L1 antibody (1:100, ab205921, Abcam), anti-CD3 antibody (1:25, ab17143, Abcam), anti-CD8 antibody (1:50, ab17147, Abcam), and anti-CD20 antibody (1:50, ab78237, Abcam). Bound antibodies were visualized with the appropriate secondary antibodies (1:500, Alexa Fluor 488, Alexa Fluor 594, Invitrogen, Waltham, Massachusetts, USA) at 37°C for 30 min at 1:500 dilution with 5% goat serum. 4’,6-diamidino-2-phenylindole (Vector Laboratories, Burlingame, California, USA) was used as a counterstain. The green fluorescence produced by Alexa 488, red fluorescence produced by Alexa 594, and blue fluorescence produced by 4’,6-diamidino-2-phenylindole were observed and captured using a fluorescence microscope BZ-X800 (Keyence, Osaka, Japan). TLSs were identified as clusters of 10 or more CD20-positive cells surrounded by CD3-positive cells approximately half the circumference, and the number of TLSs in a section was counted. The fluorescence intensities of NY-ESO-1 (CTAG1B), XAGE-1, and PD-L1 were calculated from binarized images after channeling into red, blue, and green using ImageJ software (NIH, Bethesda, Maryland, USA) from 10 randomly selected fields as previously described (11). The values higher than the mean of 62 samples of 31 cases were defined as “high”, and those lower were defined as “low”. CD8-positive cells were counted in several locations with a high infiltrating cell density. Samples with at least one CD8-positive cell in a 200x high-power field (0.40mm^2^ field area) were defined as ‘CD8-positive’, as previously described (4).

### Statistical analysis

NGS data were analyzed on the software application Transcriptome Analysis Console and Gene spring analysis (Thermo Fisher Scientific). A clustered heatmap of all samples was generated using the online tool iDEP.94 (http://bioinfomatics.sdstate.edu/idep/). A paired t-test was used to compare primary and recurrent lesions in immunohistochemistry results. Disease-specific survival was calculated as the time elapsed from definite diagnosis to death from CAS and analyzed using the Kaplan-Meier method and log-rank test. Statistical analyses were performed using Graph Pad Prism 8 (Graph Pad Software, San Diego, CA, USA). Probability values (p-values) of less than 0.05 were considered statistically significant.

## Results

### Comparison of gene expression with and without TLS

**Figure 1:**
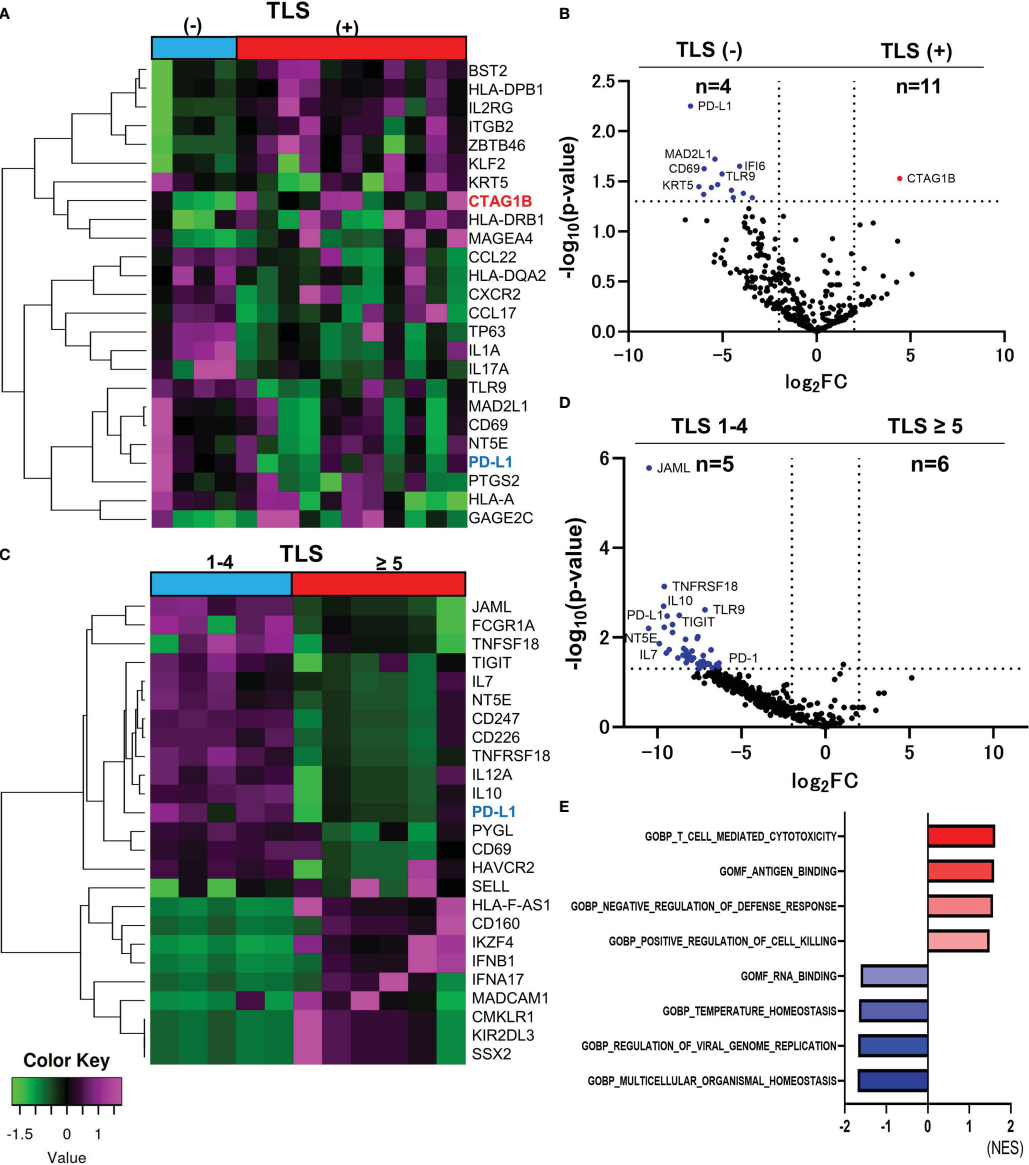
Comparison of RNA sequencing among groups classified by the number of TLSs in CAS. The heatmap of analyzed top 25 DEGs between TLS-positive and TLS-negative group (Total n=395 DEGs) **(A)**. Volcano plot indicating upregulated genes (red) and downregulated genes (blue) in TLS-positive (n=11) versus TLS-negative group (n=4) **(B)**. Heatmap of analyzed top 25 DEGs between TLS≥5 and TLS 1-4 group (Total n=395 DEGs) **(C)**. Volcano plot indicating no upregulated genes and some downregulated genes (blue) in TLS≥5 group (n=6) versus TLS 1-4 group (n=5). The vertical and horizontal broken lines represent threshold of log_2_ FC (−2 and +2) and p-value (0.05) **(D)**. The top four ranked gene sets with highest and smallest NES with a p value<0.05 in GSEA compared to TLS-positive and TLS-negative group **(E)**.

The characteristics and the immunochemical statuses of the 15 samples are summarized in **Table 1**. Hierarchical cluster analysis of the top 25 differentially expressed genes (DEGs) between TLS positive and TLS negative groups (total 395 genes) was performed (**Figure 1A**). NY-ESO-1 (CTAG1B) is the only gene that significantly upregulated in the TLS positive group (log_2_ fold-change [FC]=4.426 p-value=0.0296) (**Figure 1B**). There were no other CTAs upregulated in log_2_FC>4.4, p-value<0.1.

PD-L1 was the upregulated gene in the TLS-negative group with the smallest p-value among 395 genes (log_2_FC=-6.707, p-value=0.0006, **Figure 1B**). Hierarchical cluster analysis of top 25 DEGs between TLS≥5 and TLS=1-4 groups (total n=395 DEGs) was performed (**Figure 1C**). PD-L1 and PD-1 were upregulated in TLS=1-4 group (n=5) compared to TLS≥5 group (n=6). (PD-L1, log_2_FC=9.427, p-value=0.0033, PD-1, log_2_FC=6.335, p-value=0.0373) (**Figure 1D**). Immune checkpoint molecules seem to be deeply involved in the suppression of the TLS formation. Gene set enrichment analysis (GSEA) was performed using the Gene Ontology (GO) resource including 10, 192 gene sets. Compared between TLS-positive and TLS-negative group, 5 gene sets were upregulated in TLS-positive group, and 13 gene sets were upregulated in TLS-negative group, with a criterion of p-value < 0.05. The top four ranked gene sets with the highest and smallest normalized enrichment scores (NES) with a p-value<0.05 are shown in **Figure 1E**. ‘T cell-mediated cytotoxicity’ set, ‘Antigen binding’ set, ‘Negative regulation of defense response’ set, and ‘Positive regulation of cell killing’ set were upregulated in TLS positive group, suggesting immune activation.

### Other cancer-testis antigens were upregulated in recurrent lesions of CAS

**Figure 2:**
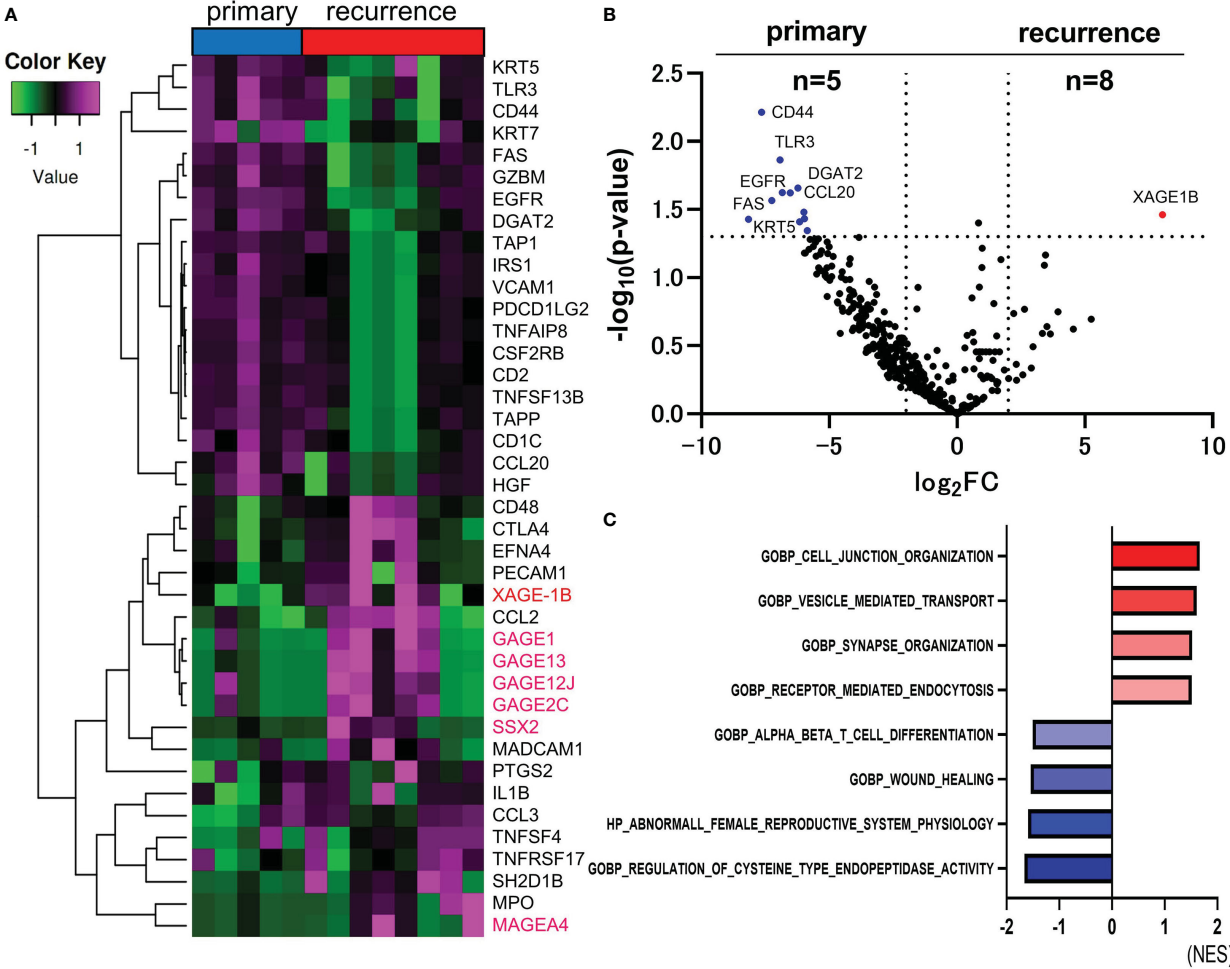
Comparison of RNA sequencing between primary and recurrent lesions in CAS. The heatmap of analyzed top 40 DEGs between primary and recurrent lesions (Total n=395 DEGs). Genes encoding CTAs are marked in magenta **(A)**. Volcano plot indicating upregulated genes (red) and downregulated genes (blue) in recurrent (n=8) versus primary lesions (n=5). The vertical and horizontal broken lines represent threshold of log_2_ FC (−2 and +2) and p-value (0.05) **(B)**. The top four ranked gene sets with highest and smallest NES with a p-value <0.05 in GSEA compared to primary and recurrent lesions **(C)**.

For comparison of the primary and recurrent lesions, hierarchical cluster analysis between primary and recurrent lesions was performed. The top 40 DEGs in the primary or recurrent lesions are shown in **Figure 2A**. CTAs, including XAGE-1B, MAGEA4, GAGE1, GAGE2C, GAGE12J, GAGE13, and SSX2 gene were upregulated in the recurrent lesions. Only XAGE-1B gene was significantly upregulated in the recurrent lesions compared to primary lesions. (log_2_ FC=8.042, p-value=0.0346) (**Figure 2B**). Total of 12 genes, such as CD44, TLR3, EGFR, FAS, and CCL20, were significantly upregulated in the primary lesions. The top four ranked gene sets with the highest and smallest NES with a p-value<0.05, which were revealed by GSEA, were shown in **Figure 2C**. CTAs accounted for 7 of the top 20 genes that were upregulated in recurrent lesions. However, their role remains unclear.

### Intra-patient heterogeneity of immune status of CAS

**Figure 3:**
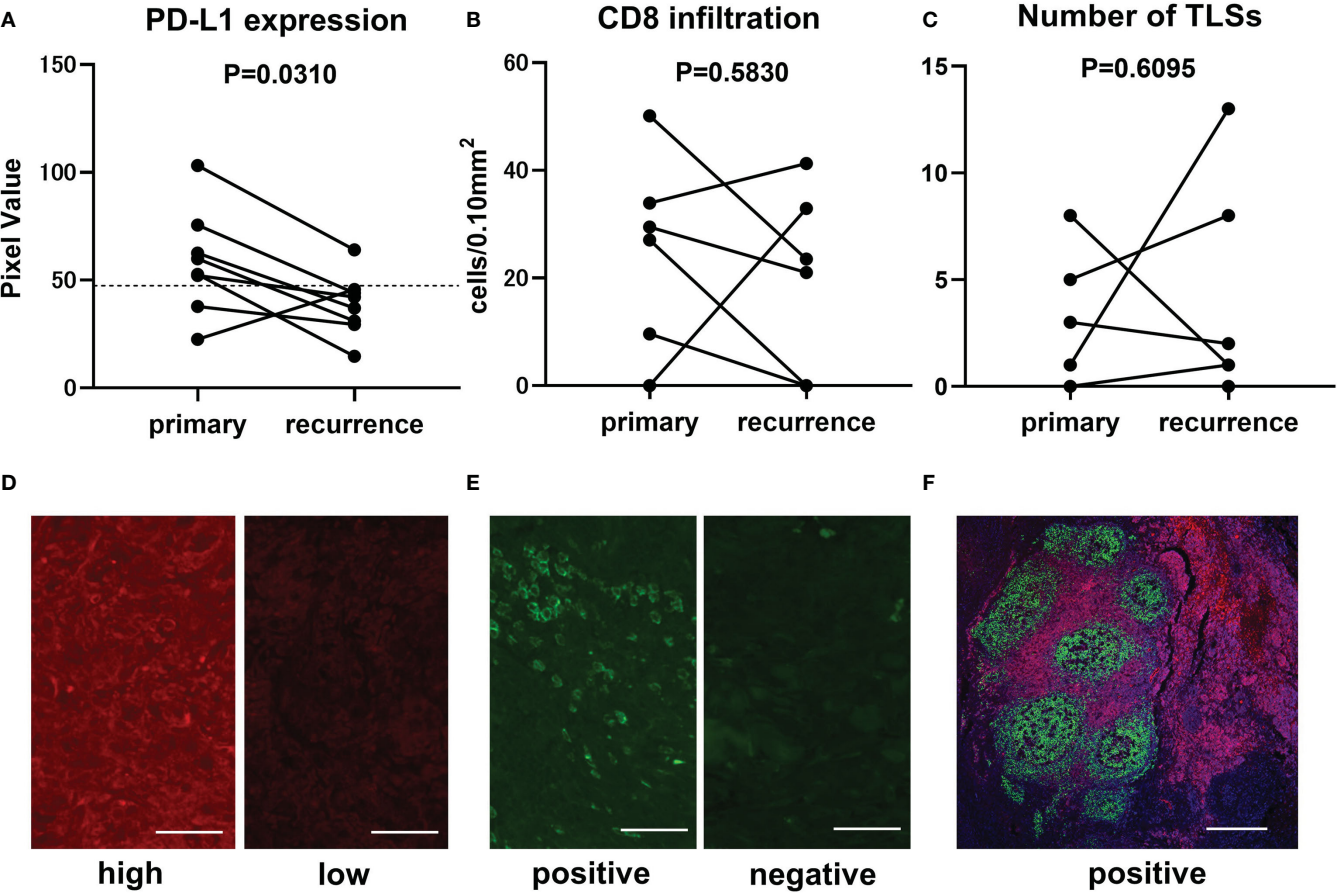
Comparison of immunofluorescence staining results between primary and recurrent lesions in CAS. Transitional change of PD-L1 expression from primary lesion to recurrent lesion is represented in the graph (n=8, paired t-test, p-value=0.0310). Mean pixel value of PD-L1 expression intensity of 62 samples was 47.4 and is indicated by broken line **(A)**. Transitional change of infiltrating CD8-positive cells (n=8, paired t-test, p-value=0.5830). The number of counted CD8-positive cells converted per 0.10 mm^2^ field area **(B)**. Transitional change of the number of TLSs in a section (n=8, paired t-test, p-value=0.6095) **(C)**. Representative immunofluorescence staining in samples with high (left panel, PV=103.2) and low (right panel, PV=37.1) PD-L1 expression. Scale bar, 50µm **(D)**. Representative immunofluorescence staining in samples with positive (left panel, 50.12 cells/0.10mm^2^) and negative (right panel, 0 cells/0.10mm^2^) CD8-positive cells infiltration. Scale bar, 50µm **(E)**. Representative immunofluorescence staining for CD20 (green), CD3 (red), and DAPI (blue) in a sample with TLSs. Scale bar, 500µm **(F)**.

Eight patients who had both primary and recurrent lesions sampled were selected to analyze intra-patient heterogeneity of potential biomarkers with immunohistochemical staining. The first recurrent lesion was picked up in a patient with multiple recurrent lesions. Biomarkers, including PD-L1 expression, the number of infiltrating CD8-positive cells, and the number of TLSs have changed dramatically in the recurrent lesions from the primary lesions. PD-L1 expression in recurrent lesions was significantly suppressed compared to primary lesions (paired t-test, p-value=0.0310) (**Figure 3A**). There was no significant tendency in the number of CD8-positive cells (paired t-test, p-value=0.5830) (**Figure 3B**) or TLSs (paired t-test, p-value=0.6095) (**Figure 3C**). The number of TLS varied widely in two cases, but only one case changed from nonexistent to present, and none of the cases had TLS disappeared later. Representative immunostaining images for PD-L1 (**Figure 3D**), CD8 (**Figure 3E**), and TLSs (**Figure 3F**)were shown.

### Chemokines associated with NY-ESO-1 and XAGE-1 expression

**Figure 4:**
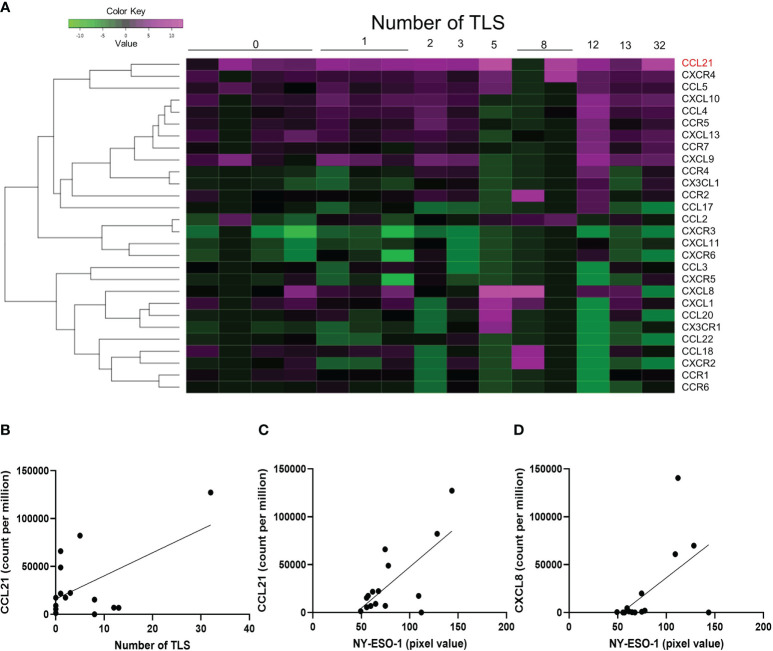
Chemokines associated with NY-ESO-1 and XAGE-1 expression. The heatmap shows the number of TLS formations and the expression of chemokines. 28 chemokines included in the Oncomine Immune Response Research Assay were analyzed **(A)**. Linear regression analysis showed that only CCL21 was correlated with the number of TLS (p-value=0.0273) **(B)**. Expression of NY-ESO-1 was correlated with CCL21 (p-value=0.0030) **(C)** and CXCL8 (p-value=0.0242) **(D)**.

Additional analysis was performed on 28 chemokines included in the Oncomine Immune Response Research Assay. The number of TLS and the expression levels of chemokines analyzed by clustering analysis for each sample are shown as a heat map (**Figure 4A**). The only chemokine correlated with the number of TLS was CCL21 (liner regression, p-value=0.0273, **Figure 4B**). Two chemokines, CCL21 (p-value=0.0037) and CXCL8 (p-value=0.0242), positively correlate with NY-ESO-1 expression in immunohistochemical staining (**Figures 4C, D**). And no chemokines correlated with XAGE-1 expression.

### Immunostaining for cancer-testis antigens

**Figure 5:**
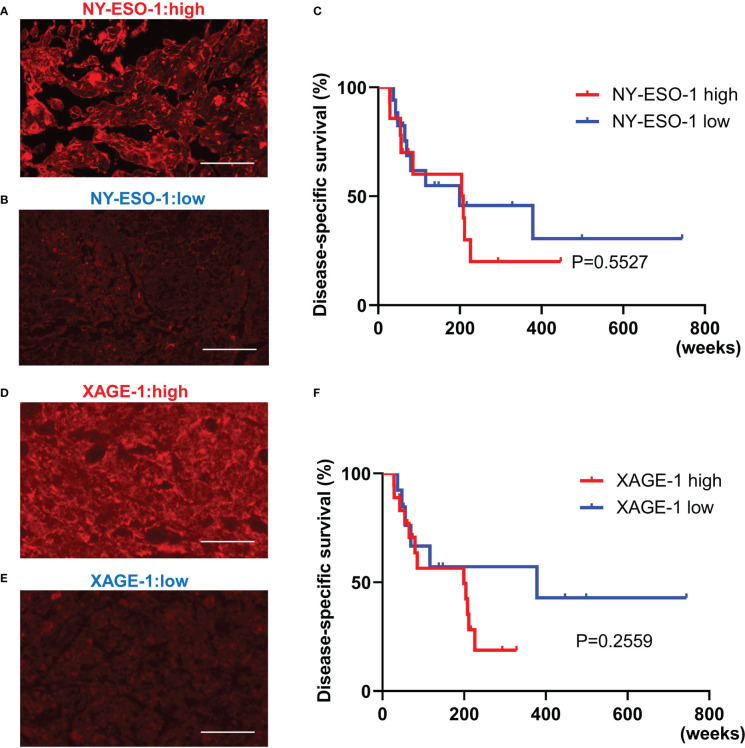
The relationship between NY-ESO-1/XAGE-1 expression in primary lesion and prognosis in CAS. Representative immunofluorescence staining in samples with high NY-ESO-1 expression. Scale bar, 50µm **(A)**. Representative immunofluorescence staining in samples with low NY-ESO-1 expression. Scale bar, 50µm **(B)**. Kaplan-Meier survival curves for the patients with high and low NY-ESO-1 expression in primary lesion using log-rank test (n=31, log-rank, p-value=0.5527). NY-ESO-1 high: 14 cases. NY-ESO-1 low: 17 cases **(C)**. Representative immunofluorescence staining in samples with high XAGE-1 expression. Scale bar, 50µm **(D)**. Representative immunofluorescence staining in samples with low XAGE-1 expression. Scale bar, 50µm **(E)**. Kaplan-Meier survival curves for the patients with high and low XAGE-1 expression in primary lesion using log-rank test (n=31, log-rank, p-value=0.2559). XAGE-1 high: 18 cases, XAGE-1 low: 13 cases **(F)**.

**Figure 6:**
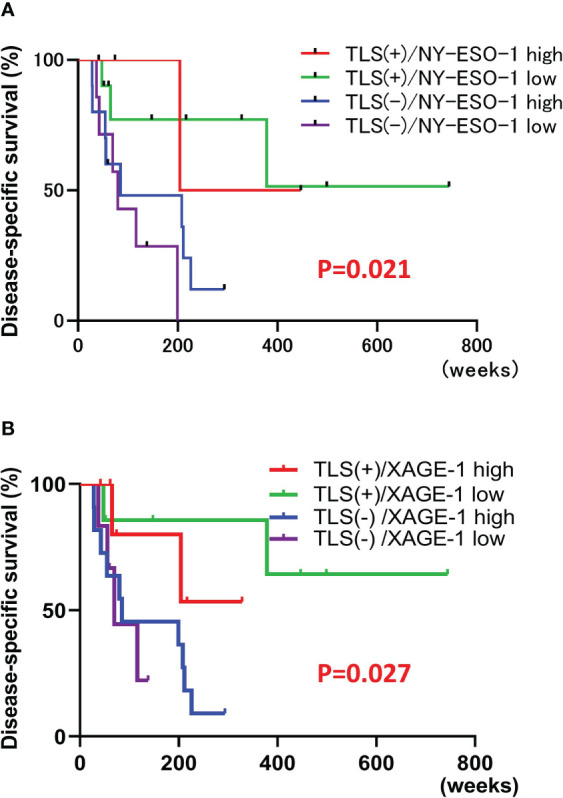
Correlation of each CTAs and TLS combination with prognosis. Kaplan-Meier survival curves for the patients classified by combination of NY-ESO-1 expression and the presence of TLSs in primary lesion using log-rank test (n=31, log-rank, p-value=0.021). TLS+/NY-ESO-1 high: 4 cases, TLS+/NY-ESO-1 low: 10 cases, TLS-/NY-ESO-1 high: 10 cases, TLS-/NY-ESO-1 low: 7 cases **(A)**. Kaplan-Meier survival curves for the patients classified by combination of XAGE-1 expression and the presence of TLSs in primary lesion using log-rank test (n=31, log-rank, p-value=0.027). TLS+/XAGE-1 high: 7 cases, TLS+/XAGE-1 low: 7 cases, TLS-/XAGE-1 high: 11 cases, TLS-/XAGE-1 low: 6 cases **(B)**.

NY-ESO-1 and XAGE-1 are detectable by immunohistochemistry. NY-ESO-1 pixel values greater than 74.8 (average of 62 samples) were defined as “high” (14 cases) and those less than 74.8 were defined as “low” (17 cases). Representative images of each are shown in **Figures 5A, B**. Kaplan-Meier curve showed that expression of NY-ESO-1 in the primary lesions does not significantly correlate with the prognosis (n=31, log-rank, p-value=0.5527) (**Figure 5C**). XAGE-1 pixel values greater than 98.2 were defined as “high (18 cases) and those less than 98.2 were defined as “low” (13 cases). Representative images of each are shown in **Figures 5D, E**. Kaplan-Meier curve showed that expression of XAGE-1 in the primary lesions does not significantly correlate with the prognosis (n=31, log-rank, p-value=0.2559, **Figure 5F**). These CTAs were significantly different in each group only when stratified by presence or absence of TLS. The combination of TLS and NY-ESO-1 showed no difference in high or low NY-ESO-1 expression in TLS-positive cases, but the NY-ESO-1 positive group tended to have a better prognosis in TLS-negative cases (n=31, log-rank, p-value=0.021, **Figure 6A**). In the combination of TLS and XAGE1, the prognosis was better in the group of TLS-positive cases with low XAGE1 levels (n=31, log-rank, p-value=0.027, **Figure 6B**).

## Discussion

The comprehensive RNA analysis in CAS revealed a significant upregulation of NY-ESO-1 in TLS-positive samples, an association of immune checkpoint molecules with suppression of TLSs, and significant upregulation of XAGE-1B in recurrent lesions. The formation of TLS and NY-ESO-1 expression correlated with a chemokine CCL21 expression. Although immunohistochemical staining results for NY-ESO-1 and XAGE-1 alone did not correlate with prognosis, their combination with TLS provided detailed prognostic information.

NY-ESO-1 and XAGE-1B are one of the CTAs, which are a large family of tumor-associated antigens only in germ cells of the testis and placenta (12, 13), and in various malignant tumors, such as melanoma (14), squamous cell carcinoma (15), non-small cell lung cancer (16), gastric cancer (17), hepatocellular carcinoma (18), breast cancer (19), ovarian cancer (20), bladder cancer (21), prostate cancer (22), multiple myeloma (23), synovial sarcoma (24), and Ewing’s sarcoma (25). CTAs can be divided into two groups, those that are encoded on the X chromosome (CT-X antigens) and those that are not (non-X CT antigens) (13). NY-ESO-1 and XAGE-1 genes are classified into CT-X antigens, which are highly expressed in tumors and have strong immunogenicity (13). CTAs contribute significantly to tumor cell physiology and affect tumor behavior, promoting tumorigenesis and antagonizing mechanisms of tumor suppression (26, 27). Several reports have suggested that CTAs were associated with tumor progression and poor prognosis (15, 17, 20–22). In patients with hepatocellular carcinoma, elevated serum XAGE-1B tends to be associated with a higher recurrence rate, and may be useful as a prognostic biomarker (18). Similarly, also in melanoma, lack of NY-ESO-1 and XAGE-1B in metastatic lymph nodes prolonged overall survival (14). CAS has been reported to be positive by immunostaining for MAGEA4 and NY-ESO-1 (28), which are CTAs, but whether or not XAGE-1 is expressed in CAS and its usefulness as a prognostic factor have not been clarified. This investigation revealed CTAs, including XAGE-1, were upregulated in CAS, especially in recurrent lesions, and only XAGE-1 expression did not correlate with prognosis, but the combination with TLSs correlates with prognosis in CAS. CTAs, which can be regarded as cancer-specific antigens due to limited expression in germ cells that are immune privileged because of their lack of HLA class I (29), are promising targets for immunotherapy. Tumor mutation burden (TMB), which is defined as the number of somatic mutations per coding area in a cancer genome and increases expression of neoantigens, is a predictor of the effect of immunotherapy (30) and positively correlate with expression of CTAs in pan-cancers (31). Although CAS is generally regarded as immunologically cold, CAS arising in the face and scalp has been found to have a higher TMB than other carcinomas such as melanoma (32). In this study, gene expression results of CAS showed that NY-ESO-1 is highly expressed in CAS cases have activated anti-tumor immunity with TLSs. In such cases, immunotherapy including ICIs should be highly effective. And CTAs, especially NY-ESO-1, may be an excellent biomarker to predict the response to ICIs. Compared to neoantigens, which arise from somatic mutations and vary from patient to patient, CTAs may be more useful as biomarkers or therapeutic targets because their expression is largely determined by disease.

It is also interesting to note that the expression of NY-ESO-1 correlates with the expression of the chemokine CCL21. CCL21 is expressed in high endothelial venules (HEVs) or lymphatic endothelial cells (LECs) and is involved in TLS formation by mediating lymphocyte mobilization (33, 34). It makes sense that CCL21 expression would be increased in CAS, tumors of blood vessels and lymph vessels. CCL21 has been reported to correlate with favorable prognosis in colorectal cancer (35) and Ewing sarcoma (36). The correlation of CCL21, TLS, and NY-ESO-1 expression is observed in this study is a useful finding that suggests a high immunosensitivity of CAS.

However, in patients with non-small cell lung cancer, high serum antibody against NY-ESO-1 and XAGE-1 levels were associated with higher efficacy of anti-PD-1 antibody therapy but did not correlate with the intensity of NY-ESO-1 and XAGE-1 immunostaining (17). In recurrent CAS lesions of this study, whereas RNA of CTAs was upregulated and the presence of TLSs was unchanged except for one case, PD-L1 expression was downregulated in immunofluorescence staining, suggesting that anti-tumor immunity is not functioning and ignores tumor. From these results, rather than tumor-side factors such as high or low expression of CTAs, host-side factors, abilities to appropriately produce antibodies against these antigens may be more important. TLSs are the frontline of tumor immunity, where antigen presentation is carried out (37). To exert anti-tumor immunity and enhance the therapeutic effect of ICIs, a tumor microenvironment in which TLS is present is desirable (38), and the focus is on creating such a situation. In the present study in CAS, the expression of PD-L1 and PD-1 was increased in the group with TLS compared to the group without TLS and in the group of TLS poor compared to the group of TLS rich, suggesting that inhibition of the PD-1/PD-L1 pathway may increase the number of TLS in CAS, as reported previously in other cancers (38) where ICI treatment increases the density of TLS.

In addition to ICI therapy, CTAs have recently been attracting attention as promising candidates for tumor vaccines and T cell therapy targets, genetically modifying T cells to express antigen-specific T cell receptors or chimeric antigen receptors (CAR) (39–41). Furthermore, expression of CTAs in tumors, including XAGE-1, is regulated by DNA methylation, one of the epigenetics, and its inhibition has been found to induce CTAs only in tumor cells (42). Therefore, methylation inhibitors are expected to enhance the therapeutic effects of various immunotherapies. Several clinical trials on the above various therapies and their combination have been conducted and are expected to be a new therapeutic strategy. In conclusion, we suggested that CAS, like other carcinomas for which immunotherapy is effective, expresses strong immunogenic CTAs and has TLSs which provide appropriate anti-tumor immunity, and this combination may be useful in predicting prognosis. Although the small sample size is the limitation of this study, we hope this report will help in considering the efficacy of immunotherapy and selecting treatments for CAS, which has a very poor prognosis and few effective treatments.

## Data availability statement

The datasets presented in this study can be found in online repositories. The names of the repository/repositories and accession number(s) can be found below: https://www.ncbi.nlm.nih.gov/, GSE203215.

## Ethics statement

The studies involving human participants were reviewed and approved by Clinical Research Management Center, Nagoya City University Hospital. Written informed consent for participation was not required for this study in accordance with the national legislation and the institutional requirements.

## Author contributions

TM and MN contributed to conception and design of the study, performed experiments and statistical analysis, wrote the first draft of the manuscript. YN and MY performed experiments. SK and HK contributed case accumulation. AM contributed to supervision and manuscript revision. All authors contributed to the article and approved the submitted version.

## Supplementary material

Supplementary Table 1 Characteristics, treatment, and immunofluorescence staining results for all patients
