# Supplementary material for: Tumor immune microenvironment of cutaneous angiosarcoma with cancer testis antigens and the formation of tertiary lymphoid structures
Source: Front Oncol. 2023 Apr 4;13:1106434. doi: 10.3389/fonc.2023.1106434 (PMC10112511; doi:10.3389/fonc.2023.1106434)
Supplement: Supplementary Table 1 — Characteristics, treatment, and immunofluorescence staining results for all patients [file Table_1.docx]

| Characteristics | | Value |
| --- | --- | --- |
| Cases |  | 31 |
| Samples |  | 62 |
|  | Primary lesion | 47 (31 cases) |
|  | Recurrent lesion | 15 (8 cases) |
| Age (range) | | 76.81 (60-95) |
| Sex |  |  |
|  | Male | 21 (67.7%) |
|  | Female | 10 (32.3%) |
| Primary Site | | cases (n=31) |
|  | Head&Neck | 26 (83.9%) |
|  | Trunk | 1 (3.2%) |
|  | Extremity | 4 (12.9%) |
| Metastases (at diagnosis) | | Cases (n=7) |
|  | Lymph node metastsis | 2 |
|  | Distant metastasis | 0 |
| Differentiation | | Samples (n=15) |
|  | Well-differentiated | 19 |
|  | Moderately differentiated | 5 |
|  | Poorly differentiated | 7 |
| Treatment |  | cases (n=31) |
|  | Surgery | 16 (51.6%) |
|  | Radiation therapy | 26 (83.9%) |
|  | Interleukin-2 | 3 (9.7%) |
|  | Taxanes | 23 (74.2%) |
|  | Eribulin | 7 (22.6%) |
|  | Pazopanib | 8 (25.8%) |
| PD-L1 expression | | samples (n=62) |
|  | Higher than average (47.4 pv) | 24 |
|  | Lower than average | 38 |
| CD8 infiltration | | samples (n=62) |
|  | Positive | 33 |
|  | Negative | 29 |
| The number of TLSs | | samples (cases) |
|  | 0 | 33 (16 cases) |
|  | 1-4 | 17 (7 cases) |
|  | 5-9 | 6 (4 cases) |
|  | 10 or more | 6 (6 cases) |
| pv, pixel value | |  |
